# Protective Effects of Hemp (*Cannabis sativa*) Root Extracts against Insulin-Deficient Diabetes Mellitus In Mice

**DOI:** 10.3390/molecules28093814

**Published:** 2023-04-29

**Authors:** Yujeong Kim, Wonhee Kim, Soo-Hyun Kim, Kyu-Sang Sim, Ki-Hyun Kim, Kiu-Hyung Cho, Gi-Seok Kwon, Jung-Bok Lee, Jun-Ho Kim

**Affiliations:** 1 Department of Food Science and Biotechnology, Andong National University, Andong 36729, Republic of Korea; pre0227@naver.com (Y.K.); 2450rla@naver.com (W.K.); 2 Life Science Team, Kyochon F&B Co., Ltd., Osan 18150, Republic of Korea; hyunsk0513@gmail.com (S.-H.K.); sim9612@naver.com (K.-S.S.); 3 Department of Research Project, Gyeongbuk Institute for Bioindustry, Andong 36618, Republic of Korea; kkh051400@gib.re.kr (K.-H.K.); khcho68@gib.re.kr (K.-H.C.); 4 Department of Horticulture & Medicinal Plant, Andong National University, Andong 36729, Republic of Korea; gskwon@andong.ac.kr; 5 Research Institute of Food & Bio, BHNBIO Co., Ltd., Jincheon-gun 27850, Republic of Korea

**Keywords:** hemp root, cannabis sativa, diabetes, islet function, β-cell apoptosis

## Abstract

The pharmacological potential of industrial hemp (*Cannabis sativa*) has been widely studied. However, the majority of studies have focused on cannabidiol, isolated from the inflorescence and leaf of the plant. In the present study, we evaluated the anti-diabetic potential of hemp root water (HWE) and ethanol extracts (HEE) in streptozotocin (STZ)-induced insulin-deficient diabetic mice. The administration of HWE and HEE ameliorated hyperglycemia and improved glucose homeostasis and islet function in STZ-treated mice (*p* < 0.05). HWE and HEE suppressed β-cell apoptosis and cytokine-induced inflammatory signaling in the pancreas (*p* < 0.05). Moreover, HWE and HEE normalized insulin-signaling defects in skeletal muscles and apoptotic response in the liver and kidney induced by STZ (*p* < 0.05). Gas chromatography-mass spectrometry analysis of HWE and HEE showed possible active compounds which might be responsible for the observed anti-diabetic potential. These findings indicate the possible mechanisms by which hemp root extracts protect mice against insulin-deficient diabetes, and support the need for further studies geared towards the application of hemp root as a novel bioactive material.

## 1. Introduction

There is a growing interest in the medical application of industrial hemp (*Cannabis sativa*), containing a high level of cannabidiol (CBD). The majority of hemp studies have focused on high purity extraction of cannabinoids, including CBD, and assessing their pharmacological activities [1,2]. In addition to cannabinoids, hemp is a rich source of other bioactive phytochemicals, including terpenoids (>120), flavonoids (>26), and steroids (>11) [3,4]. A previous study investigating the chemical profile of different parts of hemp reported that cannabinoids and flavonoids were relatively abundant in inflorescences and leaves, whereas stem barks and root contained high amounts of triterpenoids and sterols [3]. As such, several studies have recently reported the pharmacological potential of hemp root. Various compounds, including cannabinoids, triterpenoids, phytosterols, polyphenols, and fatty acids, have been identified as possible bioactive constituents in hemp root [5,6,7]. However, there is currently a lack of in vivo data demonstrating the biological and pharmacological activities of hemp root extract.

It is well established that pancreatic β-cell failure and islet dysfunction are the main features of both type 1 and type 2 diabetes [8]. The β-cells are particularly expugnable to reactive oxygen species (ROS), because of their low levels of antioxidant enzymes [9]. High glucose levels in diabetes increase the generation of ROS, which activates both the intrinsic and extrinsic apoptotic pathways in β-cells [10,11]. It has been suggested that apoptosis is the main form of β-cell death in both types of diabetes [12]. In addition to ROS-mediated cellular signaling, cytokine-induced nuclear factor–kappa B (NF-κB) and mitogen-activated protein kinase (MAPK) pathways have been shown to trigger β-cell apoptosis [12]. The increase in β-cell apoptosis by MAPK includes the activation of c-Jun N-terminal kinase (JNK), p38, and extracellular signal-regulated kinase (ERK).

Recent studies have demonstrated the superior antioxidant and anti-inflammatory potential of hemp root ethanolic extract or its bioactive compounds [5,13]. These findings suggest that hemp root extract might have protective effects on pancreatic islet function in diabetes. Therefore, this study aims to investigate the protective effects of hemp root extract against β-cell apoptosis and islet dysfunction as well as possible action mechanisms in streptozotocin (STZ)-induced insulin-deficient diabetic mice. In addition, gas chromatography-mass spectrometry (GC-MS) analysis was performed to identify possible compounds responsible for the observed effects.

## 2. Results

### 2.1. Hemp Root Extracts Protect Mice against STZ-Induced Hyperglycemia and Islet Dysfunction

**Figure 1:**
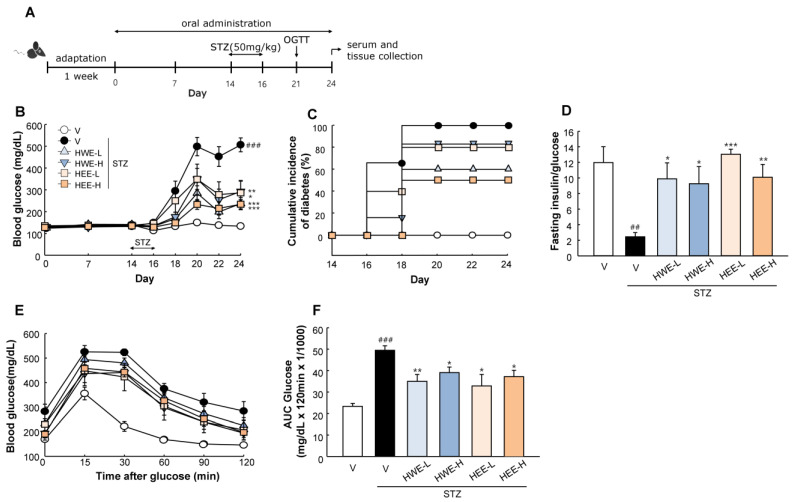
Effects of hemp root extracts on glucose homeostasis. (**A**) Timeline of the study. (**B**) Blood glucose concentrations during experimental period. (**C**) Cumulative incidence of diabetes was calculated as the percentage of hyperglycemic mice (glucose level ≥ 250 mg/dL) at each time point. (**D**) The ratio of fasting plasma insulin (pg/mL) to blood glucose (mg/dL) was used as an index of insulin deficiency in mice. (**E**) Glucose concentrations during oral glucose tolerance test with (**F**) corresponding area under the curve (AUC) measured at Day 21. V, vehicle; HWE-L, hemp root water extract at low dose; HWE-H, hemp root water extract at high dose; HEE-L, hemp root ethanol extract at low dose; HEE-H, hemp root ethanol extract at high dose. Data are shown as means ± SEM (*n* = 6–7). ^##^ *p* < 0.01, ^###^ *p* < 0.001 vs. non-STZ vehicle group; * *p* < 0.05, ** *p* < 0.01, *** *p* < 0.001 vs. STZ-vehicle group.

Figure 1A shows a timeline of the experiment. Diabetes was induced by STZ at a dose of 50 mg/kg/day for three consecutive days. Daily oral administration of HWE and HEE was initiated before 2 weeks of diabetes induction and continued until the end of the experiment. Before diabetes induction, there were no changes in blood glucose levels by HWE or HEE. After multiple STZ treatments, the administration of HWE and HEE suppressed progressive hyperglycemia (*p* < 0.05) and the cumulative incidence of diabetes compared to the STZ vehicle group (Figure 1B,C). The fasting insulin to glucose ratio, an index of insulin resistance [14], was markedly reduced following STZ treatment, but an improvement was noted upon treatment with HWE and HEE (Figure 1D). Moreover, following an oral glucose load, HWE and HEE showed an improved glucose tolerance in STZ-treated mice (*p* < 0.05) (Figure 1E,F).

**Figure 2:**
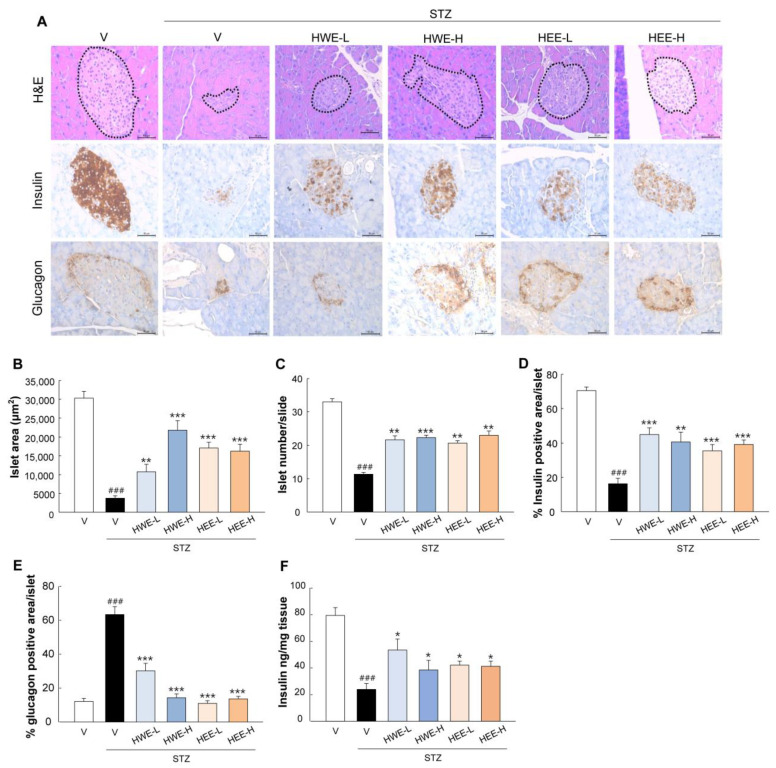
Protective effects of hemp root extracts against islet dysfunction. (**A**) H&E and DAB staining for insulin and glucagon were performed to analyze histopathological changes in pancreatic islets. H&E-stained sections were analyzed for measurements of (**B**) islet area and (**C**) islet number using ImageJ software (Version 1.8.0). Quantitative data relating to (**D**) insulin and (**E**) glucagon signals were calculated by dividing the area of target signal by the total islet area, for at least 10 islets per mouse. (**F**) The pancreatic insulin content was measured by an ELISA kit. V, vehicle; HWE-L, hemp root water extract at low dose; HWE-H, hemp root water extract at high dose; HEE-L, hemp root ethanol extract at low dose; HEE-H, hemp root ethanol extract at high dose. Data are shown as means ± SEM (*n* = 4 for (**B**–**E**) and *n* = 6 for (**F**)). ^###^ *p* < 0.001 vs. non-STZ vehicle group; * *p* < 0.05, ** *p* < 0.01, *** *p* < 0.001 vs. STZ-vehicle group.

Histological examination showed that HWE and HEE counteracted STZ-induced islet deterioration. This finding was supported by an improved islet size and number, as well as the signal distribution of insulin-producing β-cells and glucagon-producing α-cells as compared to the STZ-vehicle group (*p* < 0.01) (Figure 2A–E). These results were also confirmed by an increase in pancreatic insulin content in STZ-induced diabetic mice treated with HWE or HEE (Figure 2F).

### 2.2. Hemp Root Extracts Inhibit Pancreatic β-Cell Apoptosis and Cytokine-Induced Inflammatory Response

**Figure 3:**
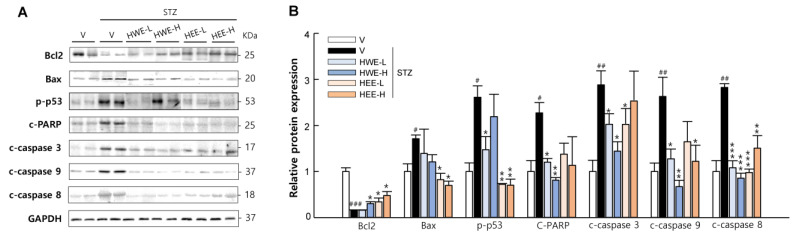
Protective effects of hemp root extracts against β-cell apoptosis. (**A**) Representative western blot images of pancreatic Bcl2, Bax, phospho-p53, cleaved PARP, cleaved caspase-3, cleaved caspase-9, and cleaved caspase-8 and (**B**) corresponding quantitative data were used for evaluating β-cell apoptosis. V, vehicle; HWE-L, hemp root water extract at low dose; HWE-H, hemp root water extract at high dose; HEE-L, hemp root ethanol extract at low dose; HEE-H, hemp root ethanol extract at high dose. Data are shown as means ± SEM (*n* = 4). ^#^ *p* < 0.05, ^##^ *p* < 0.01, ^###^ *p* < 0.001 vs. non-STZ vehicle group; * *p* < 0.05, ** *p* < 0.01, *** *p* < 0.001 vs. STZ-vehicle group.

Pancreatic β-cell apoptosis is central to the pathogenesis of type 1 and type 2 diabetes [12]. To assess whether hemp root extracts could improve islet function by preventing STZ-induced β-cell apoptosis, we analyzed the pancreatic expression levels of major proteins involved in intrinsic (mediated by mitochondria and caspase-9) and extrinsic (mediated by death receptor and caspase-8) apoptosis pathways. We observed a significant increase in β-cell apoptosis in STZ-vehicle mice, as reflected by an increased protein expression of B-cell lymphoma 2 (Bcl-2)-associated X protein (Bax), phosphorylated p53, cleaved poly (ADP-ribose) polymerase (PARP), and cleaved caspases (caspase-3, 9, and 8), and a reduced expression of Bcl-2 (*p* < 0.05) (Figure 3). However, HWE and HEE suppressed these apoptotic changes in the pancreas (*p* < 0.05).

**Figure 4:**
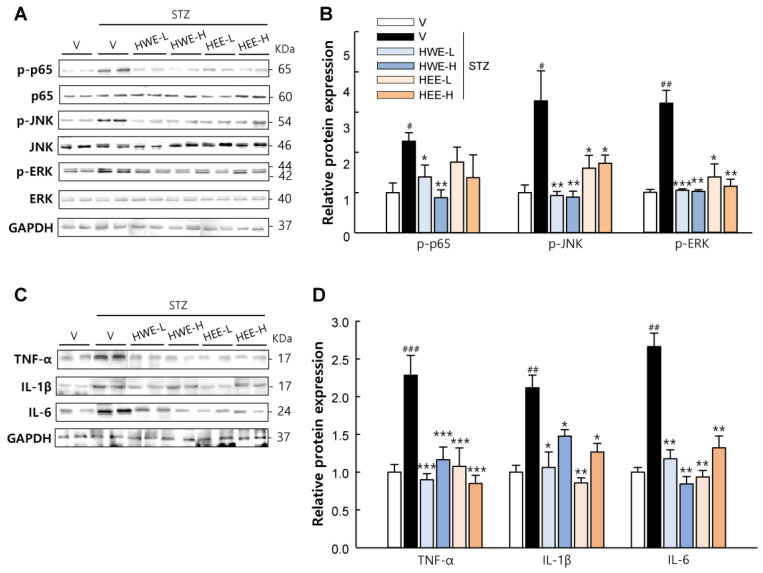
Inhibitory effects of hemp root extracts on inflammatory signaling in the pancreas. (**A**) Representative western blot images of phosphorylated-p65, p65, phosphorylated-JNK, JNK, phosphorylated-ERK, and ERK in the pancreas and (**B**) corresponding quantitative data. (**C**) Representative western blot images of TNF-α, IL-1β, and IL-6 in the pancreas and (**D**) corresponding quantitative data. V, vehicle; HWE-L, hemp root water extract at low dose; HWE-H, hemp root water extract at high dose; HEE-L, hemp root ethanol extract at low dose; HEE-H, hemp root ethanol extract at high dose. Data are shown as means ± SEM (*n* = 4). ^#^ *p* < 0.05, ^##^ *p* < 0.01, ^###^ *p* < 0.001 vs. non-STZ vehicle group; * *p* < 0.05, ** *p* < 0.01, *** *p* < 0.001 vs. STZ-vehicle group.

Since inflammatory cytokines act as key promoters of β-cell apoptosis [15], we also examined the changes in inflammatory responses in the pancreas. Our results revealed that STZ treatment increased the pancreatic expression levels of inflammatory cytokines (TNF-α, IL-1β, and IL-6) and the phosphorylation levels of p65 (a subunit of NF-κB), JNK, and ERK, which are key mediators of cytokine-induced β-cell apoptosis [12] (Figure 4). Interestingly, the expression of these inflammatory proteins was effectively suppressed following HWE or HEE treatment (*p* < 0.05). Thus, these results suggest that islet protection by HWE and HEE might be associated with the inhibition of cytokine-induced and NF-κB-/MAPK-mediated apoptotic signaling in β-cells.

### 2.3. Hemp Root Extracts Attenuate Apoptosis in Liver and Kidney and Improve Insulin Signaling in Skeletal Muscle

**Figure 5:**
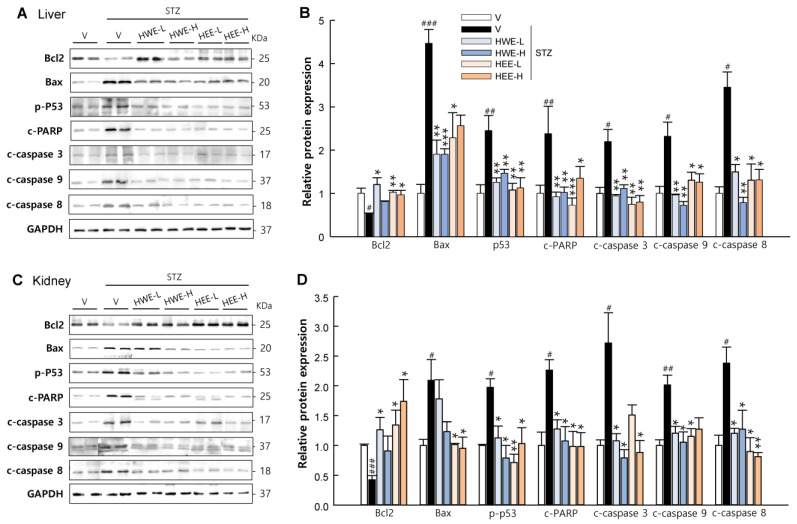
Inhibitory effects of hemp root extracts on apoptotic signaling in the liver and kidney. Representative western blot images of Bcl2, Bax, phospho-p53, cleaved PARP, cleaved caspase-3, cleaved caspase-9, and cleaved caspase-8, and corresponding quantitative data in the liver (**A**,**B**) and kidney (**C**,**D**). V, vehicle; HWE-L, hemp root water extract at low dose; HWE-H, hemp root water extract at high dose; HEE-L, hemp root ethanol extract at low dose; HEE-H, hemp root ethanol extract at high dose. Data are shown as means ± SEM (*n* = 4). ^#^ *p* < 0.05, ^##^ *p* < 0.01, ^###^ *p* < 0.001 vs. non-STZ vehicle group; * *p* < 0.05, ** *p* < 0.01, *** *p* < 0.001 vs. STZ-vehicle group.

**Figure 6:**
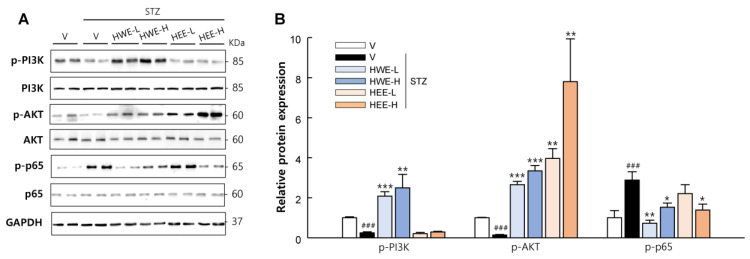
Effects of hemp root extracts on insulin signaling in skeletal muscle. (**A**) Representative western blot images of phosphorylated-PI3K, PI3K, phosphorylated-AKT, AKT, phosphorylated-p65, and p65 in the skeletal muscle and (**B**) corresponding quantitative data. V, vehicle; HWE-L, hemp root water extract at low dose; HWE-H, hemp root water extract at high dose; HEE-L, hemp root ethanol extract at low dose; HEE-H, hemp root ethanol extract at high dose. Data are shown as means ± SEM (*n* = 4). ^###^ *p* < 0.001 vs. non-STZ vehicle group; * *p* < 0.05, ** *p* < 0.01, *** *p* < 0.001 vs. STZ-vehicle group.

In addition to the protective effects of hemp root extracts in the pancreas, we also monitored their effect on the liver, kidney, and on skeletal muscle. As expected, HWE and HEE effectively suppressed both intrinsic and extrinsic apoptosis signaling induced by STZ treatment in the liver and kidney (*p* < 0.05) (Figure 5). Phosphoinositide-3-kinase (PI3K) and protein kinase B (AKT) act as key mediators for insulin-stimulated glucose uptake in skeletal muscle [16]. The phosphorylation levels of PI3K and AKT were markedly reduced in STZ-vehicle mice but significantly increased in HWE- or HEE-treated mice (*p* < 0.01), except PI3K in the HEE group (Figure 6). Moreover, the phosphorylation level of p65, which is known to induce glucose metabolism abnormalities and muscle wasting [17], was normalized in STZ-induced diabetic mice following HWE and HEE treatment (*p* < 0.05).

### 2.4. Compound Identification in Hemp root Extracts by Gas Chromatography-Mass Spectrometry

GC-MS chromatograms of HWE and HEE are shown in Figure S1. Those identified compounds with >70% of similarity based on mass spectral library are listed in Table 1. According to previous studies [18,19,20,21,22,23,24,25,26,27,28,29,30,31,32,33,34,35,36,37], 8 identified compounds in HWE and 9 compounds in HEE have been reported to exhibit antidiabetic, anti-inflammatory, or antioxidant properties based on in vitro and in vivo models. Among these compounds, vanillin, apocynin, methyl palmitate, and syringaldehyde have received considerable attention for their multiple pharmacological properties.

## 3. Discussion

The majority of studies on the therapeutic application of industrial hemp have focused on the biological activities of CBD. This study is the first to demonstrate the ability of hemp root extracts to counteract STZ-induced diabetes in mice by preventing pancreatic islet dysfunction and insulin-signaling defects in muscle. Our study reports the beneficial effects of hemp root, which has been considered a minor part of the plant with trace amounts of CBD.

The most important finding of this study is that HWE and HEE effectively suppressed hyperglycemia and protected the pancreas from islet dysfunction by inhibiting STZ-induced β-cell apoptosis. Moreover, these protective effects were associated with the suppression of inflammatory cytokines production and subsequent NF-κB and MAPK activation. In autoimmune diabetes, leukocyte infiltration and secretion of pro-inflammatory cytokines, such as TNF-α and IL-1β, act as critical factors in β-cell failure [38]. It has been suggested that these pro-inflammatory cytokines could promote both intrinsic and extrinsic apoptotic pathways in β-cells [39,40]. These apoptotic responses are predominantly mediated by the activation of NF-κB, MAPK, and p53 and their regulation of β-cell gene networks [12,41]. In this study, we observed that hemp root extracts suppressed the phosphorylation of p65, JNK, ERK, and p53 in the pancreas as well as intrinsic and extrinsic pro-apoptotic markers. Therefore, these results suggest that hemp root extracts could counteract STZ-induced β-cell failure by suppressing cytokine-induced inflammatory signaling. To more clearly elucidate the anti-diabetic action of hemp root extracts, its regulatory effects on ROS-mediated signal transduction need to be examined in β-cells and/or diabetic rodent models.

HWE and HEE attenuated apoptosis in the liver and kidney as well as improving insulin signaling in the skeletal muscle. STZ is taken up by target cells via glucose transporter 2 (GLUT2) in the plasma membrane and causes cell death (apoptosis and necrosis) via multiple mechanisms. Thus, apart from pancreatic β-cells, hepatocytes and renal tubular cells, which also express GLUT2, are susceptible to STZ [42]. Furthermore, it has been shown that STZ-induced hyperglycemia promotes skeletal muscle atrophy with increased p65 phosphorylation and impaired insulin signaling [43,44]. NF-κB and AKT play critical roles in muscle protein degradation and synthesis and thus have been considered as potential targets for treating diabetes-induced skeletal muscle atrophy [44,45]. Findings from this study showed that STZ treatment increased apoptotic protein expressions in the liver and kidney and inhibited the PI3K/AKT pathway in the skeletal muscle with increased p65 phosphorylation. However, these responses were mostly reversed by co-treatments of HWE or HEE with STZ, suggesting the potential protective effects of hemp root extracts against diabetic complications.

GC-MS analyses revealed that several compounds could be responsible for the anti-diabetic effects of HWE and HEE (Table 1). Vanillin, apocynin, methyl palmitate, and syringaldehyde, identified in HWE and HEE, have been shown to display multiple biological activities including anti-diabetic, antioxidant, and anti-inflammatory properties [22,23,25,26,27,30,31,32,33]. To our knowledge, the presence of these compounds in other plant parts of hemp has not been identified. Vanillin has been found to decrease serum glucose and improve insulin sensitivity as well as liver and renal functions in STZ-induced diabetic rats [46]. In addition, apocynin and syringaldehyde have been shown to protect experimental animals against STZ-induced hyperglycemia and insulin resistance [47,48]. These results indicate that phenolic compounds such as vanillin, apocynin, and syringaldehyde could be responsible for the anti-diabetic effects of hemp root extracts. In addition to these phenolic compounds, other bioactive compounds, including triterpenoids, phytosterols, and fatty acids as well as cannabinoids, have been identified in hemp root [5,6,7]. Therefore, further studies to fractionate hemp root extracts could provide more information for understanding the anti-diabetic potential of hemp root.

## 4. Materials and Methods

### 4.1. Sample Preparation

Hemp (*Cannabis sativa* L.) cultivated in Andong-si, Gyeongsangbuk-do, Republic of Korea was harvested in July 2022. The roots were separated from the whole plants and dried. The dried material was ground and extracted with 10 volumes of distilled water at 60 °C for 10 h or with 70% ethanol at room temperature for 3 h. Extracts were then filtered, concentrated under reduced pressure, and freeze-dried. Prepared samples were stored at 4 °C until further use.

### 4.2. Animal Study

Male C57BL/6J mice at 7 weeks old (Orient Bio Inc., Seongnam-Si, Republic of Korea) were housed with 12 h light–dark cycles and fed a standard pellet diet during the experimental period. All mice were acclimatized under laboratory conditions for 1 week and randomly divided into six treatment groups: (1) non-STZ + vehicle, (2) STZ + vehicle, (3) STZ + hemp root water extract (HWE) at 150 mg/kg (HWE-L), (4) STZ + HWE at 300 mg/kg (HWE-H), (5) STZ + hemp root ethanol extract (HEE) at 150 mg/kg (HEE-L), and (6) STZ + HEE at 300 mg/kg (HEE-H). The water and ethanol extracts were dissolved in saline containing 2% Tween-80 and 0.5% methylcellulose, and administered once daily by gavage using an esophageal cannula. After 2 weeks of oral administration, diabetes was induced by intraperitoneal injection of STZ dissolved in 50 mM citrate buffer (pH 4.5) at a dose of 50 mg/kg/day for three consecutive days. The control group received only citrate buffer. At the end of the study, mice were euthanized using an overdose of avertin (2,2,2-tribromoethanol). Blood was collected by cardiac puncture and centrifuged at 15,000× *g* at 4 °C for 20 min to collect serum samples. Pancreas, liver, kidney, and skeletal muscles were isolated for immunohistochemical staining and molecular analysis. Figure 1A shows a timeline of the experiment. All animal work was carried out in strict accordance with the institutional guidelines for the use and care of laboratory animals. The study protocol was approved by the Ethical Committee of Andong National University (Protocol Number: 2022-1-0228-01-01).

### 4.3. Glucose and Insulin Measurements

Blood glucose levels in tail blood samples were measured before and after STZ injection using a glucometer (OneTouch Ultra 2, LifeScan, Inc., Milpitas, CA, USA). The cumulative incidence of diabetes was calculated as the percentage of hyperglycemic mice (non-fasting blood glucose level ≥ 250 mg/dL) per treatment group at each time point. For the oral glucose tolerance test (OGTT), mice were given glucose (2 g/kg body weight) by oral gavage after a 16 h fast. Blood glucose levels were monitored at indicated time points before and after glucose administration. The area under the curve (AUC) for glucose during OGTT was calculated for each experimental group. Plasma insulin levels were determined using an ELISA kit (Millipore Co., Billerica, MA, USA) following the manufacturer’s instructions.

The pancreas was homogenized in acidified ethanol and incubated for 16 h at 4 °C. Pancreas extracts were centrifuged at 3000× *g* for 10 min at 4 °C. The insulin content of the supernatant was determined using an ELISA kit (Millipore).

### 4.4. Histology and Immunostaining

The isolated pancreas was fixed in 10% neutral-buffered formalin, dehydrated using a graded series of alcohol, and embedded in paraffin. Sections (2 μm in thickness) were deparaffinized, rehydrated, and stained with hematoxylin and eosin (H&E). Four H&E-stained sections per mouse were used for measuring islet area using ImageJ software (Version 1.8.0, National Institutes of Health, NIH, Bethesda, MA, USA). For microscopy imaging of insulin and glucagon, deparaffinized pancreas sections were incubated with primary antibodies (Abcam, Cambridge, UK) overnight at 4 °C. Staining was visualized using mouse-specific HRP/DAB detection IHC kit (Abcam). Images of islets were acquired using a microscope (Leica Microsystems, Wetzlar, Germany). The percentage of target signal-positive area was calculated by dividing the area of target signal by the total islet area for at least 10 islets per mouse.

### 4.5. Western Blot Analysis

Pancreas, liver, kidney, and skeletal muscle tissues were homogenized in a lysis buffer containing phenylmethylsulfonyl fluoride (Roche, Mannheim, Germany) and a protease inhibitor cocktail (Sigma-Aldrich, St. Louis, MO, USA) to prepare protein lysates. The total protein concentration was determined using the Bradford method. Equal amounts of protein were separated on 12% SDS/PAGE and transferred to polyvinylidene difluoride membranes. The membranes were blocked for 30 min in PBS containing 3% BSA and 0.1% Tween-20 for 1 h at room temperature under constant agitation. These membranes were then probed with the primary antibodies listed in Table S1, followed by incubation with corresponding horseradish peroxidase-conjugated secondary antibodies (Sigma-Aldrich). The protein bands were visualized using enhanced chemiluminescence reagents using a Fusion Solo 6S EDGE imaging system (Vilber, Marne-la-Vallée, France) and quantified using the ImageJ software (Version 1.8.0, NIH).

### 4.6. Gas Chromatography-Mass Spectrometry Analysis

Bioactive compounds in hemp root extracts were identified using the GC-MSD system (Agilent 5977A, Santa Clara, CA, USA). These compounds were separated using a DB-5MS column at a flow rate of 1 mL/min. The oven was operated at 50 °C for 5 min, followed by heating at 5 °C/min to 250 °C, and holding for 5 min. The sample (1 μL) was injected into the column in the split mode (10:1). The identification of compounds was carried out using the National Institute of Standards and Technology 11 mass spectral library. Compounds matched with >70% of similarity are listed in Table 1.

### 4.7. Statistical Analyses

All statistical analyses were performed using one-way ANOVA with the post hoc Tukey HSD method using R software (4.0.4 for Windows). Results are expressed as mean ± SEM. *p* values < 0.05 were considered as statistically significant.

## 5. Conclusions

In conclusion, the present study demonstrated that HWE and HEE counteracted STZ-induced hyperglycemia and islet dysfunction via the inhibition of β-cell apoptosis in mice. The inhibition of β-cell apoptosis by HWE and HEE was associated with the suppression of cytokine-induced inflammatory signaling. In addition, HWE and HEE attenuated apoptosis in the liver and kidney and improved insulin signaling in skeletal muscle. These findings provide novel scientific evidence for the pharmaceutical application of hemp root, which has been considered a minor part of the plant in *Cannabis*-based medicinal and functional food studies.

## Figures and Tables

**Table 1:** Bioactive compounds identified in hemp root extracts by GC-MS.

Sample	Retention Time	Compound Name	Molecular Formula	Similarity (%) Matched with Library	Peak Area (%)	Reported Biological Activities	Reference
HWE	14.136	Guaiacol	C_7_H_8_O_2_	87	2.920	Antioxidant	[18]
	20.459	5H-1-Pyrindine	C_8_H_7_N	81	1.803	–	
	20.957	4-Vinylguaiacol	C_9_H_10_O_2_	93	7.913	Antioxidant, Anti-inflammatory	[19]
	21.938	Syringol	C_8_H_10_O_3_	97	3.270	Antioxidant, Anti-inflammatory	[20,21]
	23.26	Vanillin	C_8_H_8_O_3_	91	0.645	Antidiabetic, Antioxidant, Anti-inflammatory	[22,23]
	24.598	Chavibetol	C_10_H_12_O_2_	93	1.785	Antioxidant	[24]
	25.462	Apocynin	C_9_H_10_O_3_	76	1.370	Anti-diabetic, Antioxidant, Anti-inflammatory	[25,26,27]
	26.471	Deamino-oxo 4-methylthioamphetamine	C_10_H_12_OS	72	1.652	–	
	30.505	2-Hydroxy-4-isopropyl-7-methoxytropone	C_11_H_14_O_3_	90	2.332	–	
	31.305	Coniferyl alcohol	C_10_H_12_O_3_	89	4.940	Antioxidant, Anti-inflammatory	[28,29]
	35.271	Methyl palmitate	C_17_H_34_O_2_	93	1.363	Andi-diabetic, Antioxidant, Anti-inflammatory	[30,31,32]
HEE	14.137	Mequinol	C_7_H_8_O_2_	87	0.925	–	
	18.299	2,3-Dihydrobenzofuran	C_8_H_8_O	80	47.141	–	
	20.953	4-Vinylguaiacol	C_9_H_10_O_2_	91	10.735	Antioxidant, Anti-inflammatory	[19]
	21.952	Syringol	C_8_H_10_O_3_	93	2.056	Antioxidant, Anti-inflammatory	[20,21]
	23.265	Vanillin	C_8_H_8_O_3_	91	1.805	Antidiabetic, Antioxidant, Anti-inflammatory	[22,23]
	26.477	Deamino-oxo 4-methylthioamphetamine	C_10_H_12_OS	72	1.891	–	
	29.525	Syringaldehyde	C_9_H_10_O_4_	76	1.482	Antidiabetic, Antioxidant, Anti-inflammatory	[33]
	31.12	Acetosyringone	C_10_H_12_O_4_	83	0.674	Antioxidant	[34]
	31.305	Coniferyl alcohol	C_10_H_12_O_3_	96	6.537	Antioxidant, Anti-inflammatory	[28,29]
	35.951	Palmitate	C_16_H_32_O_2_	95	7.248	Anti-diabetic	[35]
	36.601	Ethyl palmitate	C_17_H_34_O_2_	95	0.894	Anti-inflammatory	[31]
	39.109	Linoleate	C_18_H_32_O_2_	93	0.456	Anti-diabetic, Anti-inflammatory	[36,37]

## Data Availability

The datasets used and/or analyzed during the current study are available from the corresponding author on reasonable request.

## Supplementary Materials

The following supporting information can be downloaded at: https://www.mdpi.com/article/10.3390/molecules28093814/s1, Figure S1: GC-MS chromatograms of hemp root extracts; Table S1: Antibodies used for western blotting.

## Sample Availability

Samples of the compounds are available from the corresponding author on reasonable request.
