# Supplementary material for: Protective Effects of Hemp (Cannabis sativa) Root Extracts against Insulin-Deficient Diabetes Mellitus In Mice
Source: Molecules. 2023 Apr 29;28(9):3814. doi: 10.3390/molecules28093814 (PMC10179809; doi:10.3390/molecules28093814)
Supplement: Supplementary file 1 [file molecules-28-03814-s001.zip › Table S1.docx]

**Table S1. Antibodies used for western blotting.**

| **Primary antibody** | **Clone** | **Company** | **Catalog No.** | **Dilution** |
| --- | --- | --- | --- | --- |
| **p65** | Polyclonal | Thermo Fisher | PA5-27617 | 1:1000 |
| **p-p65** | Polyclonal | ABcam | ab86299 | 1:2000 |
| **p-p53** | Polyclonal | ABcam | ab1431 | 1:1000 |
| **p38** | Polyclonal | Cell Signaling | #9212 | 1:1000 |
| **p-p38** | Polyclonal | ABcam | ab47363 | 1:2000 |
| **JNK** | Monoclonal | ABcam | ab179461 | 1:2000 |
| **p-JNK** | Monoclonal | ABcam | ab124956 | 1:2000 |
| **ERK** | Polyclonal | ABcam | ab17942 | 1:2000 |
| **p-ERK** | Monoclonal | ABcam | ab201015 | 1:2000 |
| **p-p53** | Polyclonal | ABcam | ab1431 | 1:1000 |
| **PI3K** | Monoclonal | Thermo Fisher | MA1-74183 | 1:1000 |
| **p-PI3K** | Polyclonal | Thermo Fisher | PA5-104853 | 1:1000 |
| **AKT** | Polyclonal | Cell Signaling | #9272 | 1:1000 |
| **p-AKT** | Polyclonal | Cell Signaling | #9271 | 1:1000 |
| **BAX** | Monoclonal | Cell Signaling | #2772 | 1:1000 |
| **Bcl2** | Monoclonal | ABcam | ab692 | 1:500 |
| **c-Cas3** | Polyclonal | Cell Signaling | #9661 | 1:1000 |
| **c-Cas8** | Monoclonal | Cell Signaling | #8592 | 1:1000 |
| **c-Cas9** | Polyclonal | Cell Signaling | #9509 | 1:1000 |
| **c-PARP** | Monoclonal | ABcam | ab32064 | 1:1000 |
| **TNF-α** | Polyclonal | ABcam | ab6671 | 1:2000 |
| **IL-1β** | Polyclonal | Santa Cruz | sc-7884 | 1:1000 |
| **IL-6** | Monoclonal | Cell Signaling | #12912 | 1:1000 |
| **GAPDH** | Monoclonal | Abbkine | A01020-SK | 1:2000 |
