# Supplementary figures and images for: Protective Effects of Hemp (Cannabis sativa) Root Extracts against Insulin-Deficient Diabetes Mellitus In Mice
Source: Molecules. 2023 Apr 29;28(9):3814. doi: 10.3390/molecules28093814 (PMC10179809; doi:10.3390/molecules28093814)

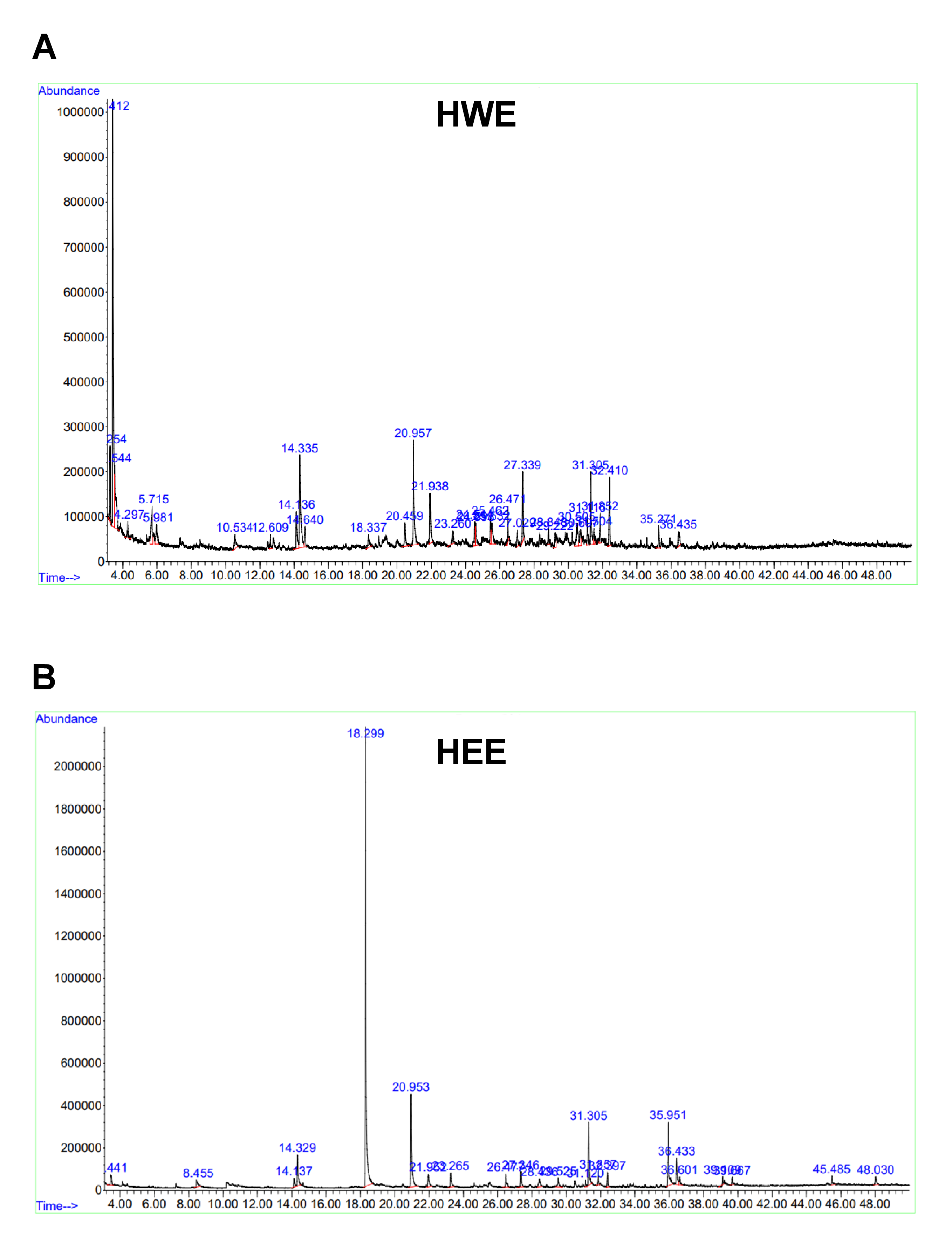

Supplement: Supplementary file 1 [file molecules-28-03814-s001.zip › Figure S1.tif]
